# Atopic dermatitis treated with tralokinumab and upadacitinib combination therapy: A case report

**DOI:** 10.1177/2050313X231180766

**Published:** 2023-06-14

**Authors:** Rafael Paolo Lansang, Irene X Zhao, Perla Lansang

**Affiliations:** 1 Michael G. Degroote School of Medicine, McMaster University, Hamilton, ON, Canada; 2 Division of Dermatology, University of Toronto, Toronto, ON, Canada

**Keywords:** Atopic dermatitis, eczema, tralokinumab, upadacitinib

## Abstract

A patient presented to clinic with atopic dermatitis that had been previously unresponsive to multiple topical and systemic therapies. They were successfully treated with a combination of tralokinumab and upadacitinib, showing significant improvement after 3 weeks and near-resolution after 6 months.

## Introduction

Biologic therapies and Janus kinase inhibitors have both been successfully used in the management of patients with atopic dermatitis.^1,2^ To our knowledge, trials investigating the combination of these therapies have not been published. We present a case of atopic dermatitis treated successfully with a combination of tralokinumab and upadacitinib.

## Case report

A 42-year-old male patient with atopic dermatitis (AD) had attempted multiple systemic therapies (Table 1), topical steroids, and calcineurin inhibitors over the past 8 years with little to no success. At baseline, the patient presented with Dermatology Life Quality Index (DLQI) = 26, extra amniotic saline infusion (EASI) = 19.4, body surface area (BSA) = 47%, and had flares in which BSA was greater than 80%. The patient showed moderate improvement when treated for 5 months with 30 mg of upadacitinib (Rinvoq) per day along with topical treatments, but the clinical response was inconsistent. Tralokinumab (Adtralza) was added to the patient’s treatment regimen. The patient showed marked improvement of the plaques and decreased pruritus within 3 weeks and almost complete resolution (BSA = 1%, EASI < 1%) after 6 months. The patient is continuing management of their AD with a combination of tralokinumab, upadacitinib, and topical treatments. As of last follow-up, the patient had been on this combination for 8 months. The patient has reported no adverse events.

**Table 1.** Systemic therapies attempted without resolution.

Systemic therapies	Duration	Outcome
Mycophenolate mofetil	Two separate 3-month courses, and one - month course of 500 mg BID	Inconsistent improvement in pruritus, worsened plaques
Cyclosporine	One 4-month course of 50–150 mg BID	Temporary improvement followed by worsening of plaques
Methotrexate	One 2-month course and one 4-month course	Improved pruritus, no effect on plaques or erythema
Azathioprine	One 10-day course	No improvement. Developed gastrointestinal adverse effects
Tralokinumab monotherapy trial	2 injections, d/c after 1 month	Worsened pruritus and plaques
Dupilumab monotherapy	30 months	Moderate improvement in plaques, significant reduction of pruritus. Flares still present. Severe ocular adverse effects
Upadacitinib monotherapy	One 1-month course and one 4-month course of 30 mg daily	Inconsistent moderate improvement in plaques. Developed folliculitis

## Discussion

The multifaceted pathophysiology of AD makes combination therapy an intriguing avenue in the management of the condition. The efficacy and tolerability seen in our patient, along with the lack of response to multiple previous monotherapies, encourages the possibility of treating unresponsive AD with biologic therapies and JAK inhibitors simultaneously.
